# Supplementary material for: AffyMAPSDetector: a software tool to characterize Affymetrix GeneChip™ expression arrays with respect to SNPs
Source: BMC Bioinformatics. 2007 Jul 30;8:276. doi: 10.1186/1471-2105-8-276 (PMC1959249; doi:10.1186/1471-2105-8-276)
Supplement: Additional file 13 — AffyMAPSDetector v1 source code. AffyMAPSDetector source code. [file 1471-2105-8-276-S13.zip › AffyMAPSDetector_v1_src/doc/jwf/package-use.html]

Uses of Package jwf


|  |  |  |  |
| --- | --- | --- | --- |
| **Overview** \| **Package** \| Class \| **Use** \| **Tree** \| **Deprecated** \| **Index** \| **Help** | | |  |
| PREV   NEXT | **FRAMES**    **NO FRAMES**     **All Classes** |  |  |


---


## **Uses of Package jwf**

|  |  |
| --- | --- |
| Packages that use jwf | |
| **affychip.snpdetector.ui** |  |
| **affychip.snpdetector.util** |  |
| **jwf** | This package contains classes that can be used to create and run a Wizard. |

Classes in jwf used by affychip.snpdetector.ui

****Wizard****
  
          This class controls a wizard.

****WizardListener****
  
          Implement this interface to be able to receive wizard related events.

****WizardPanel****
  
          The base class used for implementing a panel that is displayed in
a Wizard.

Classes in jwf used by affychip.snpdetector.util

****Wizard****
  
          This class controls a wizard.

****WizardListener****
  
          Implement this interface to be able to receive wizard related events.

Classes in jwf used by jwf

****Wizard****
  
          This class controls a wizard.

****WizardContext****
  
          An instance of this class is maintained by the Wizard.

****WizardListener****
  
          Implement this interface to be able to receive wizard related events.

****WizardPanel****
  
          The base class used for implementing a panel that is displayed in
a Wizard.

---


|  |  |  |  |
| --- | --- | --- | --- |
| **Overview** \| **Package** \| Class \| **Use** \| **Tree** \| **Deprecated** \| **Index** \| **Help** | | |  |
| PREV   NEXT | **FRAMES**    **NO FRAMES**     **All Classes** |  |  |


---
